# Prenatal ultrasound findings of ectodermal dysplasia: a case report

**DOI:** 10.1186/s12884-022-04430-7

**Published:** 2022-02-04

**Authors:** Liang Li, Yi Zhou, Ruixia Tian, Chaoxue Zhang

**Affiliations:** 1 Department of Ultrasound, The First Affiliated Hospital of Anhui Medical University, 218 Jixi Road, Hefei, 230022 Anhui China; 2 Department of Obstetrics and Gynecology, The No.901 Hospital of the Joint Service of the People’s Liberation Army, Hefei, 230022 Anhui China

**Keywords:** Ectodermal dysplasia, Prenatal ultrasound, Ectodysplasin gene, Case report

## Abstract

**Background:**

Ectodermal Dysplasia is a diverse group of inherited disorders characterized by a congenital defect in two or more ectodermal structures. Due to a fairly low incidence, to the best of our knowledge there are few clues that can assist in making an effective prenatal ultrasound diagnosis. Currently, the prenatal diagnosis of ectodermal dysplasia depends on a fetal genetic test combined with the family history. In this case report, we present a fetal case of ectodermal dysplasia with a remarkable prenatal ultrasound image, genetic testing, family history, and relevant exams of the stillbirth.

**Case presentation:**

A multipara with a 22-week singleton male pregnancy undergoing a fetal ultrasound examination. The image showed a hypoplastic maxilla and mandible. Subsequently, the ectodermal dysplasia was defined using a family history and genetic testing. The skin pathology from the aborted fetus demonstrated a hypohidrotic type. The computed tomography (CT) reconstruction after induced labor confirmed the prenatal ultrasound findings of the maxilla and mandible.

**Conclusions:**

This case suggested that prenatal ultrasound may provide a valuable clue of ectodermal dysplasia. The diagnosis can be established using further prenatal genetic testing and a family history.

## Background

Ectodermal dysplasia (ED) was first proposed by Thurman in 1848. It is a diverse group of inherited disorders characterized by a congenital defect in two or more ectodermal structures. The primary manifestations are abnormalities in hair, teeth, nails or sweat glands [1]. The inheritance mode includes autosomal dominant, autosomal recessive, X-linked dominant, and recessive. Currently, ED can be classified into the hidrotic or hypohidrotic types according to whether it involves sweat glands. Hypohidrotic ectodermal dysplasia (HED) has a birth prevalence rate of 1/50,000–100,000, and males with the disease show all or most of the typical clinical manifestations associated with the ectodysplasin A (EDA) gene, while female carriers show less severe symptoms [2]. The X-linked type is the most common form of hypohidrotic ED. Prenatal diagnosis for this rare disorder is helpful in predicting potential postpartum hyperpyrexia or providing a basis for termination of pregnancy or suggesting the possibility of prenatal correction [3, 4]. We reviewed the cases reported in the last 20 years, and a majority of the confirmed cases were diagnosed by genetic tests after birth. A prenatal tooth germ sonography has been suggested to detect X-linked hypohidrotic ED in some studies (Table 1), but it has not been used as a diagnostic basis in a broader setting. This is because not all the tooth germs could be accurately counted using ultrasound in middle pregnancy, and the risk of a false positive would probably increase unnecessary treatment [11]. The prenatal diagnosis should be confirmed by combining ultrasound findings, family history, and genetic testing [6]. Herein, we describe a rare fetal case of ED with an X-linked recessive family history that was diagnosed prenatally using ultrasound and genetic testing after diagnostic amniocentesis. Three-dimensional reconstruction CT of the induced labor fetus showed the same malformation of the alveolar as the prenatal ultrasound, and the pathological exam of the skin confirmed the hypohidrotic-type ED diagnosis.

**Table 1:** Cases of prenatal diagnosis of ectodermal dysplasia reported in PubMed in the last 20 years

Article	year of the publication	Sex	Sonographic findings	Genetic conditions	Family history	Journal
Article [4]	2018	2 males	no tooth germs at all were detected in the mandible and 1 and 2, respectively, were detected in the maxilla.	EDA c.911A → G(p.Y304C). Y304	Y	N Engl J Med
Article [5]	2020	Male	No tooth germ.	EDA c.574G	Y	Zhonghua Yi Xue Yi Chuan Xue Za Zhi
Article [6]	2014	4 males, 2 females	All 6 cases reduced number of tooth germs and 5 of them had hypoplastic lower jaws.	p. P17GfsX81 p. P220_P225del p. R156H Exon3dupl p. R156H p. P220_P225del	Y	Ultraschall Med
Article [7]	2005	Male	➖	EDA inv. (x) (p22q13)	Y	Zhonghua Yi Xue Za Zhi
Article [8]	2013	Male	➖	EDA p.G11R	Y	Zhonghua Yi Xue Yi Chuan Xue Za Zhi
Article [9]	2003	Male	a small nose and thick, everted upper and lower lips.	➖	Y	J Ultrasound Med
Article [10]	2021	Male	thinner upper alveolar bones and fewer tooth germs.	EDA Xq13.1	N	J Clin Ultrasound

## Case presentation

**Fig. 1:**
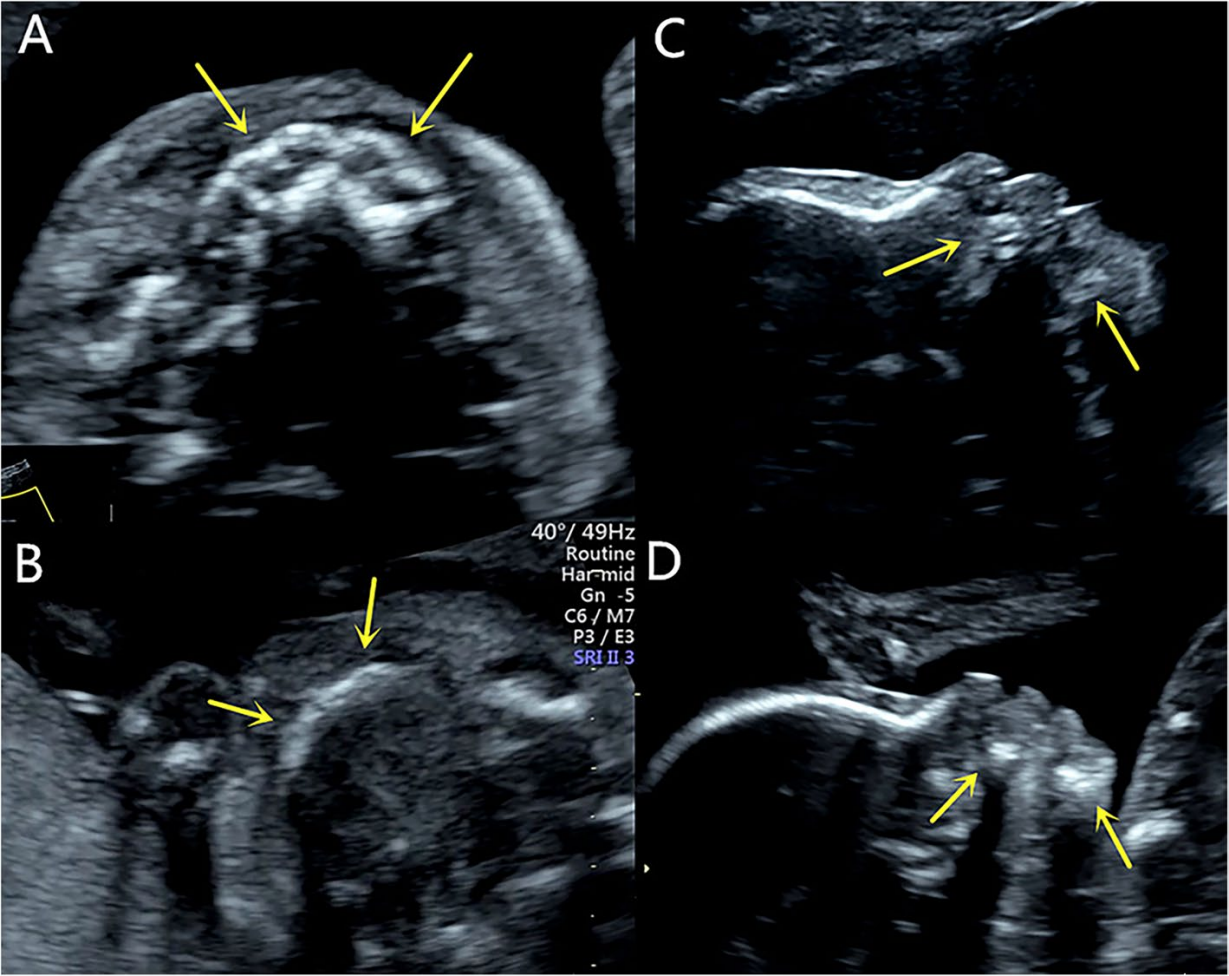
**A** A healthy control fetus of the same gestational age, the axial view image shows the normal alveolar, representing by round hypoechoic tooth germs that are arranged in an arch-like fashion in the alveolar bone. (arrow); **B** The fetus in our case, the axial view image of the maxilla shows the alveolar as a short, thin and flat hyperechoic without tooth germs (arrow); **C** A healthy control fetus of the same gestational age, the sagittal view image of the profile shows the normal maxilla and mandible (arrow); **D** The fetus in our case, the sagittal view image of the profile shows the short and retrograde maxilla and mandible (arrow)

**Fig. 2:**
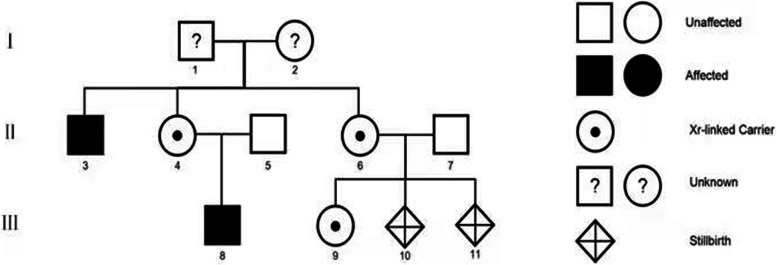
The family tree is constructed, number 11 in lineIIIis the suffered fetus in this case

**Fig. 3:**
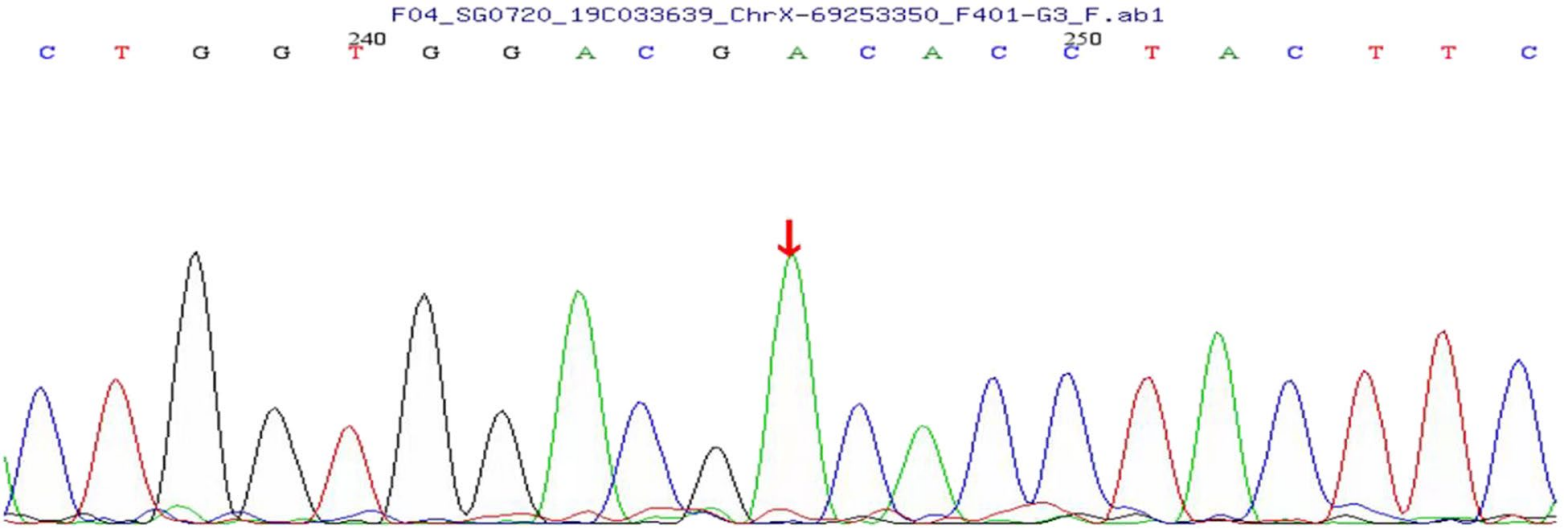
The genetic testing map shows a hemizygous variation of the EDA gene located inXq12 ~ 13.1

**Fig. 4:**
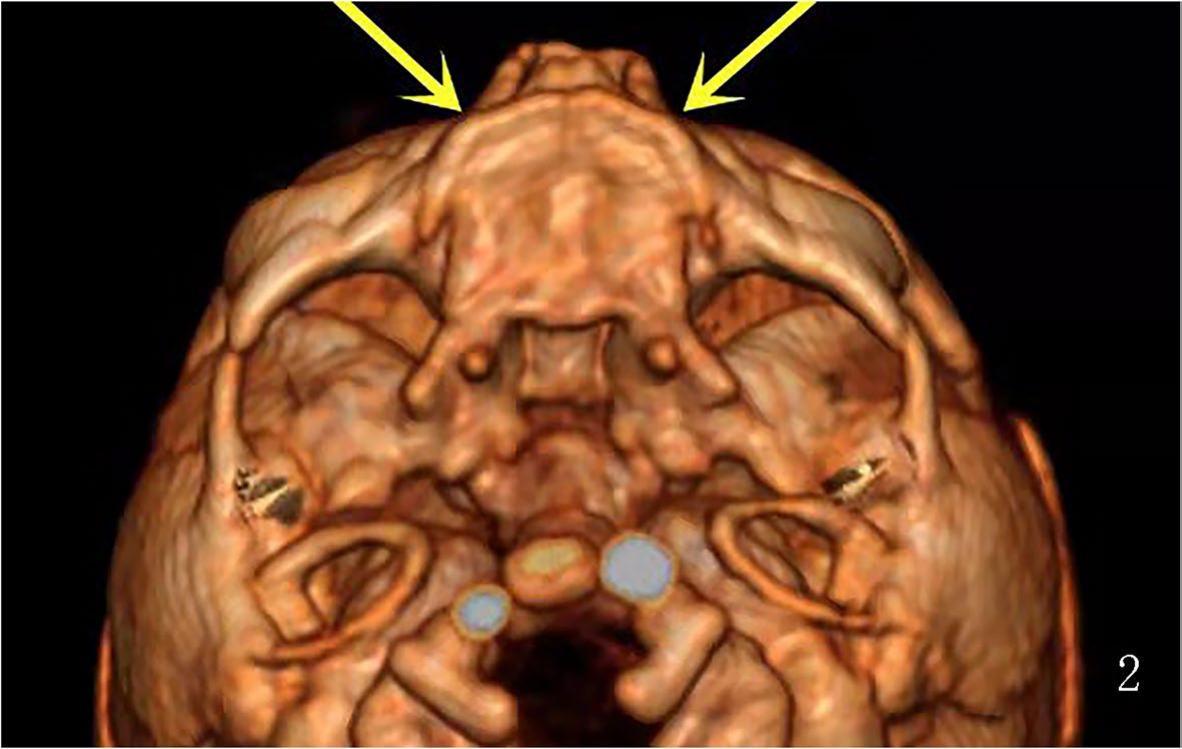
Three-dimensional CT image reconstruction of the facial bone after induced labor showing a short and flat anterior palate without tooth germ in the axial view

**Fig. 5:**
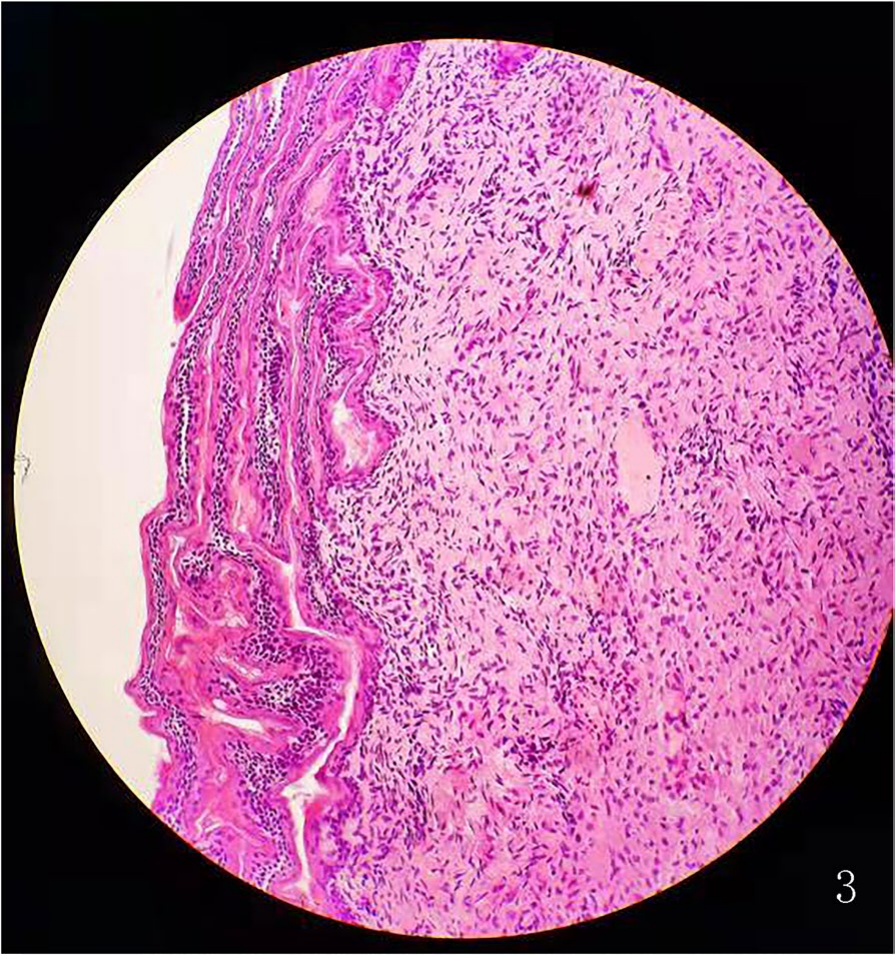
The skin histopathology: skin tissue H&E staining from the hypothenar shows no hair follicles and sweat glands in the dermis

A 29-year-old multipara, gravida 4, para 1, with 22-week singleton male pregnancy was referred to our center due to a family history of ED. Her primary concern was whether the fetus was diseased, and if so, she wanted to make the decision to induce labor based on the severity. An ultrasound performed in our center showed that the maxilla and the mandible were short and retrograde. In the axial view, no hypoechoic tooth germs were detected in both the upper and lower alveolar bones. The alveolar bone was obviously thin and hypoplastic (Fig. 1). Three-dimensional imaging in the transparent mode did not show the abnormalities of the alveolar as the gray-scale images, and no unusual facial features were found in the surface mode. After genetic counseling, we learned that the pregnant woman’s older brother had abnormal maxillary and mandible development, no teeth, and thin hair. The pregnant woman’s nephew, seven-years old, also suffered from similar symptoms. The pregnant women’s first born is now a six-year old girl and completely normal. The second pregnancy resulted in spontaneous abortions in the first trimester. During the third pregnancy at 5 months, the women received a fetal genetic detection that confirmed a hemizygous variation of the EDA located in Xq12–13.1. The variation of the same gene was also detected in the pregnant woman’s diseased brother and nephew. This was consistent with the X-linked recessive inheritance characteristics (Fig. 2). Due to the ultrasound findings and the genetic testing, the parents decided to terminate the third pregnancy. In this report, it was her forth pregnancy. After the ultrasound exam, the pregnant woman underwent diagnostic amniocentesis. The Sanger sequencing demonstrated the EDA gene mutation (C.896G > A, p.Gly299Asp), which is a known pathogenic gene (Fig. 3). The parents decided to terminate the pregnancy based on the ultrasound findings and genetic testing. We performed a three-dimensional reconstruction CT of the induced labor fetus. The images showed that the upper and lower alveolar ridges were flat and straight. No tooth germs were detected. The maxilla and mandible were dysplastic (Fig. 4). We collected five skin samples from different parts of the induced-labor fetal body. The pathological exams showed no hair follicles and sweat glands of the dermis in all five samples (Fig. 5).

## Discussion and conclusions

This was a rare case of ED with a characteristic prenatal ultrasound image. The patient had a family history that was consistent with the X-linked recessive inheritance characteristics. For the two male family members and the first induced male fetus of this pregnant women, the genetic testing confirmed the mutations of the X-chromosomal gene ectodysplasin A that leads to developmental defects of the hair, teeth, and various endocrine glands. The primary postpartum clinical concern for ED is the inability to sweat, and this places the affected individuals at risk for hyperthermia [12]. Fortunately, the two affected individuals did not suffer from obvious hyperthermia. The absence of teeth was the primary reason for them to seek medical help. For male sufferers, the severity of the pathogenic EDA variant may lead to varying degrees of symptoms. The cases of missense mutations and a small deletion of EDA are known to lead to milder symptoms. However, the definite mechanism is still unclear [13]. Thus, an ultrasound examination based on alveolar scanning may be the only reliable prenatal approach to evaluate the severity of symptoms.

Some studies have considered the ultrasound as a prenatal diagnosis approach for X-linked recessive inheritance ED using teeth germ counting, and this eliminates the significant risks associated with invasive diagnostic procedures such as amniocentesis [6]. However, the numerical threshold of the tooth germ count remains controversial. In our opinion, the duration of the examination and accuracy of tooth germ count is primarily affected by the indefinite position of the fetal head, and this may lead to an inaccurate result.

The present pregnancy was a male fetus with a typical dysplastic maxilla and mandible. In a normal fetus, the alveolar process is the protruding part of the lower margin of the maxilla and the upper margin of the mandible surrounding the tooth germs. In the gray-scale ultrasound, the characteristic images of the alveolar in the transverse view is a parallel strong echo zone that is arched and uneven, and the regular hypoechoic tooth germ structure can be observed in the two strong echo zones [14]. Compared with a fetus of the same gestational age, there was no hypoechoic tooth germ structure in this case, and the alveolar was obviously dysplastic. In addition, we also obtained an impression that the maxilla and the mandible were significantly narrow and retrograde in both the axial view and the sagittal view, but regretfully there was no reference range to follow. Some studies have reported the micrognathia in the three-dimensional image as a characteristic feature of HED [9]. However, we did not obtain this image in our case, and this may have been due to different facial characteristics due to the racial difference. For dental surgery, severe dysplasia of the alveolar bone means a poor prognosis [15]. A dysplastic mandible and alveolar may cause mastication problems and reduced nutritional intake. Failure to thrive has been observed in many affected boys [16]. These were the primary reasons the pregnant woman in our case chose to terminate the pregnancy. A three-dimensional reconstruction of the CT images after induced labor confirmed the prenatal ultrasound findings of the maxilla and the mandible. Some studies have used a histopathology analysis of skin biopsies from the hypothenar region in HED patients to show that the atrophic or immature sweat glands could be observed in the reticular dermis compared to a control group [17]. The histopathology of induced labor fetal skin showed the absence of hair follicles and sweat glands in our case, and this is a reliable predictor of hypohidrosis.

For a fetus with a specific family history, prenatal genetic testing can demonstrate a diagnosis of ED. A dysplastic maxilla and mandible accompanied by a tooth germless alveolar are also characteristic of ED in ultrasound images. So we suggest the mid-trimester ultrasound assessment of tooth germ, maxilla and mandible in high-risk pregnancies. However, we still lack approaches to predict the severity of the postnatal symptoms, especially for hypohidrosis. An ultrasound evaluation of alveolar development provides evidence to perform prenatal genetic testing, and this may even affect the pregnancy outcome. According to this case, a preimplantation genetic diagnosis in at-risk couple is also an option to avoid an abnormal pregnancy or to suggest the assisted reproductive technology.

## Data Availability

All data generated or analysed during this study are included in this published article.

## Abbreviations

ED: Ectodermal Dysplasia
EDA gene: Ectodysplasin A gene
HED: Hypohidrotic ectodermal dysplasia

## References

[1] Schneider H, Hammersen J, Preisler-Adams S, Huttner K, Rascher W, Bohring A (2011). Sweating ability and genotype in individuals with X-linked hypohidrotic ectodermal dysplasia. J Med Genet.

[2] Martínez-Romero MC, Ballesta-Martínez MJ, López-González V, Sánchez-Soler MJ, Serrano-Antón AT, Barreda-Sánchez M (2019). EDA, EDAR, EDARADD and WNT10A allelic variants in patients with ectodermal derivative impairment in the Spanish population. Orphanet J Rare Dis.

[3] Hammersen J, Wohlfart S, Goecke TW, Köninger A, Stepan H, Gallinat R (2019). Reliability of prenatal detection of X-linked hypohidrotic ectodermal dysplasia by tooth germ sonography. Prenat Diagn.

[4] Schneider H, Faschingbauer F, Schuepbach-Mallepell S, Körber I, Wohlfart S, Dick A, Schneider P (2018). Prenatal correction of X-linked hypohidrotic ectodermal dysplasia. N Engl J Med.

[5] Duan F, Wang C, Ren S, Kong X (2020). Prenatal diagnosis of a fetus with X-linked hypohidrotic ectodermal dysplasia. Zhonghua Yi Xue Yi Chuan Xue Za Zhi.

[6] Wünsche S, Jüngert J, Faschingbauer F, Mommsen H, Goecke T, Schwanitz K (2015). Noninvasive prenatal diagnosis of hypohidrotic ectodermal dysplasia by tooth germ sonography. Ultraschall Med.

[7] Shi HJ, Fang Q, Wang LT (2005). Prenatal diagnosis of X-linked anhidrotic ectodermal dysplasia with X-chromosome inversion. Zhonghua Yi Xue Za Zhi.

[8] Liu N, Shi HR, Wu QH, Jiang M, Kong XD. [Mutation analysis and first-trimester prenatal diagnosis for a Chinese family with hidrotic ectodermal dysplasia]. Zhonghua Yi Xue Yi Chuan Xue Za Zhi 2013; 30(4):407–409. Chinese. 10.3760/cma.j.issn.1003-9406.2013.04.006 23926005

[9] Sepulveda W, Sandoval R, Carstens E, Gutierrez J, Vasquez P (2003). Hypohidrotic ectodermal dysplasia: prenatal diagnosis by three-dimensional ultrasonography. J Ultrasound Med.

[10] Li TG, Ma B, Tie HX, Zhang QH, Hao SJ, Guan CL (2021). Prenatal sonographic diagnosis of X-linked hypohidrotic ectodermal dysplasia: an unusual case. J Clin Ultrasound.

[11] Seabra M, Vaz P, Valente F, Braga A, Felino A (2017). Two-dimensional identification of fetal tooth germs. Cleft Palate Craniofac J.

[12] Dall’Oca S, Ceppi E, Pompa G, Polimeni A (2008). X-linked hypohidrotic ectodermal dysplasia: a ten-year case report and clinical considerations. Eur J Paediatr Dent.

[13] Park JS, Ko JM, Chae JH (2019). Novel and private EDA mutations and clinical phenotypes of Korean patients with X-linked hypohidrotic ectodermal dysplasia. Cytogenet Genome Res.

[14] Rotten D, Levaillant JM (2004). Two and three-dimensional sonographic assessment of the fetal face.1. A systematic analysis of the normal face. Ultrasound Obstet Gynecol.

[15] Deo K, Sharma YK, Shah B, Kothari P, Chavan D, Sitaniya S (2019). Improvement in the quality of life of a patient of ectodermal dysplasia with reconstructive surgeries. J Cutan Aesthet Surg.

[16] Motil KJ, Fete TJ, Fraley JK, Schultz RJ, Foy TM, Ochs U (2005). Growth characteristics of children with ectodermal dysplasia syndromes. Pediatrics..

[17] Reyes-Reali J, Mendoza-Ramos MI, Garrido-Guerrero E, Méndez-Catalá CF, Méndez-Cruz AR, Pozo-Molina G (2018). Hypohidrotic ectodermal dysplasia: clinical and molecular review. Int J Dermatol.

